# Supplementary material for: What Can We Learn from the Previous Research on the Symptoms of Selective Mutism? A Systematic Review
Source: Behav Sci (Basel). 2025 Oct 31;15(11):1485. doi: 10.3390/bs15111485 (PMC12649584; doi:10.3390/bs15111485)
Supplement: Supplementary file 1 [file behavsci-15-01485-s001.zip › behavsci-3824759-supplementary.pdf]

Supplementary S1

Table S1. Characteristics of Studies with Normative Reference.

Study	Study Design	Sample Characteristics*			Symptom Measures**	Symptoms Reported
		N	Mean Age (SD)	% female		
Black & Uhde (1995)	Cross-Sectional	30	8.2 (2.6)	70.0	Parent Questionnaire (PQ), School Questionnaire, Teacher Rating Scale (TRS)	Anxiety, Obsessional, Separation Anxiety, Shyness, Social Anxiety, Specific Fears
Dummit et al. (1996)	Feasibility Study	21	8.2	76.2	CDI, Liebowitz Social Anxiety Scale, Parent Questionnaire, Social Behavior Scale	Fear of Negative Evaluation, Social Anxiety, Social Avoidance
Dummit et al. (1997)	Cross-Sectional	50	8.2 (2.7)	72.0	CDI, Liebowitz Social Anxiety Scale, Parent Questionnaire, Social Behavior Scale	Fear of Speaking, Social Anxiety, Social Avoidance
Kristensen (2001)	Case-Control	54	9.0 (3.4)	59.3	CBCL, TRF, YSR	Aggressive Behavior, Anxiety/Depressive Symptoms, Attention Problems, Externalizing Problems, Internalizing Problems, Social Problems, Thought Problems, Withdrawal
Bergman et al. (2002)	Prevalence Study	12	/	50.0	SASC-R, SSQ, TRF	Anxiety/Depressive Symptoms, Internalizing Problems, Social Anxiety, Withdrawal
Kristensen (2002)	Case-Control	54	9.0 (3.4)	59.3	Medical Reports, Motor Tests	Motor Skill Impairments, Tics/Stereotypies
Kristensen & Torgersen (2002)	Case-Control	26	8.6 (3.4)	73.1	EAS, Parental Ratings	Emotionality, Reduced Activity Levels, Reduced Social Activity, Shyness
Elizur & Perednik (2003)	Case-Control	19	/	47.4	CBCL, Mental Health Index	Behavioral Problems, Reduced Social Skills, Internalizing Symptoms, Irritability, Social Anxiety/Phobia, Stubbornness
Manassis et al. (2003)	Case-Control	14	10.1 (2.4)	64.0	CDI, CRS-R, LACT, MASC, PPVT-III, RCMAS, SASC	Anxiety, Social Anxiety
Yeganeh et al. (2003)	Case-Control	23	9.4 (1.9)	56.5	BAT, CBCL, FSSC-R, SPAI-C, STAI-C	Anxiety, Anxiety/Depressive Symptoms, Internalizing Problems, Reduced Social Skills, Social Anxiety, Withdrawal
Bar-Haim et al. (2004)	Case-Control	16	8.21 (3.5)	68.8	Acoustic Reflex Training	Specific Deficiencies in Auditory Efferent Activity
Cunningham et al. (2004)	Case-Control	52	7.2	59.6	Academic Performance Rating, OCHS-R, SSRS, WRAT-R	Anxiety, Obsessive-Compulsive Behaviors, Reduced Social Assertion, Reduced Social Cooperation, Reduced Social Responsibility, Somatic Complaints
McInnes et al. (2004)	Case-Control	7	9.7 (1.8)	/	Cognitive Measures, CRS–R, Language Assessment, MASC, RCMAS, Working Memory Assessment	Anxiety

Vecchio & Kearney (2005)	Case-Control	15	/	/	CBCL, Family Environment Scale, OCHS-R, Pictorial Scale of Perceived Competence, SSRS, TRF	Internalizing Problems, Reduced Social Activity
Cunningham et al. (2006)	Case-Control	58	7.1 (1.8)	55.2	OCHS-R, Pictorial Scale of Perceived Competence, SSRS	Depressive Symptoms, Generalized Anxiety, Obsessive-Compulsive Behaviors, Reduced Nonverbal Social Skills, Reduced Verbal Social Skills, Separation Anxiety, Social Anxiety, Somatic Complaints
Kristensen & Oerbeck (2006)	Case-Control	32	10.6 (3.1)	56.3	BVRT, Visual Memory Assessment, WISC-R	Reduced Auditory-Verbal Memory Span
Melfsen et al. (2006)	Case-Control	9	11.9 (2.42)	77.8	SPAI-C	Social Anxiety
Yeganeh et al. (2006)	Case-Control	21	10.5	66.7	ECBI, SPAI-C	Social Anxiety
Arie et al. (2007)	Case-Control	18	8.9 (2.0)	55.6	CBCL, SCARED-C, SCARED-P, SPAI-C	Anxiety, Differential Auditory Processing, Internalizing Problems, Social Anxiety
Manassis et al. (2007)	Case-Control	44	7.8 (1.6)	72.7	Finger Windows Test, LACT, MASC, PPVT-III, SASC-R, TROG, Visual Patterns Test, WISC	Anxiety, Language Impairments, Social Anxiety, Visual Memory Deficits
Bergman et al. (2008)	Case-Control	48	5.8 (1.7)	62.5	MASC-P, SASC-R	Social Anxiety
Cohan et al. (2008)	Cross-Sectional	130	/	66.2	CBCL, CCC-2, ECBI, SASC–R, VABS–II	Anxiety, Internalizing Problems, Social Anxiety, Syntax Problems
Letamendi et al. (2008)	Cross-Sectional	102	/	/	CBCL	Anxiety/Depressive Symptoms
Oerbeck & Kristensen (2008)	Case-Control	23	11.6 (2.7)	47.8	CBCL, Motor Tests, Trail Making Test, WISC-R	Anxiety/Depressive Symptoms, Attention Deficit, Attention Problems, Motor Skill Impairments, Withdrawal
Carbone et al. (2010)	Case-Control	44	8.2 (3.4)	52.3	Assessment of Verbal Social Skills, Nonverbal Social Skills and Nonverbal Cooperation, SCARED, SSRS	Anxiety, Generalized Anxiety, Internalizing Problems, Lower Self Control, Reduced Nonverbal Cooperation, Reduced Nonverbal Social Skills, Reduced Social Assertion, Reduced Social Cooperation, Reduced Social Responsibility, Reduced Social Skills, Reduced Verbal Social Skills, School Avoidance, Separation Anxiety, Social Anxiety
Henkin et al. (2010)	Case-Control	10	9.4 (2.6)	70	EEG-Paradigm, SCARED-P, SPAI-C	Anxiety, Differential Auditory Processing, Social Anxiety
Nowakowski et al. (2011)	Case-Control	19	6.4 (0.9)	58	Behavioral Observations	Fewer Episodes of Joint Attention
Heilman et al. (2012)	Case-Control	20	/	60	Physiological Measures (Heart Rate, Respiration, Activity)	Inflexible Physiological Stress Response
Oerbeck et al. (2012)	Feasibility Study	7	4.4 (0.8)	71.4	ASEBA, CCC-2, EAS, Stanford-Binet, PPVT-IV	Shyness, Withdrawal
Sharkey & McNicholas (2012)	Cohort Study	14	6.9	78.6	SCAS, SDQ	Anxiety, Behavioral Problems, Emotional Difficulties, Problems with Peer Interaction
Young et al. (2012)	Case-Control	10	7.0 (1.8)	60	Behavioral Assessment of Social Interaction, CBCL, Physiological Measures (Electrodermal Activity,	Anxiety, Internalizing Problems, Reduced Social Skills, Social Anxiety

					Blood Pressure, Heart Rate), SPAI-C, SPAIC-PV	
Alyanak et al. (2013)	Case-Control	26	8.1 (2.1)	57.7	BSI, CBCL	Anxiety/Depressive Symptoms, Attention Problems, Internalizing Problems, Social Problems, Thought Problems, Withdrawal
Klein et al. (2013)	Cross-Sectional	33	7.3 (1.6)	57.6	EVT-2, PPVT-4, TNL	Reduced Expressive Narrative Language Competence
Levin-Decanini et al. (2013)	Case-Control	48	6.5 (2.6)	72.9	CPRS-R:L, CTRS-R:L	Anxiety/Shyness, Social Problems
Muchnik et al. (2013)	Case-Control	31	8.9 (3.1)	70.9	Auditory Brainstem Responses (ABRs), Measures of Middle-Ear Acoustic Reflex (MEAR), Transient-evoked otoacoustic emissions (TEOAE, MOCB)	Differential Auditory Processing
Cholemkery et al. (2014)	Case-Control	43	11.1 (3.9)	40	CBCL, DISYPS-III: FBB-ADHD, DISYPS-III: FBB-ANZ, SCQ, SRS, WISC/CFT 20-R	Attention Deficit/Hyperactivity, Anxiety, Anxiety/Depression, Autistic Mannerisms, Internalizing Problems, Social Awareness Problems, Social Cognition Problems, Social Communication Problems, Social Motivation Problems, Somatic Complaints, Withdrawal
Martinez et al. (2015)	Case-Control	19	7.5 (1.5)	68.4	MASC, SASC-R	Harm Avoidance, Perfectionism, Problems Performing in Public, Social Anxiety
Gensthaler, Maichrowitz, et al. (2016)	Case-Control	95	9.7 (4.5)	50.5	CBCL, YSR	Anxiety/Depressive Symptoms, Attention Problems, Internalizing Problems, Social Problems, Withdrawal
Esposito et al. (2017)	Randomized Controlled Trial	166	7.81 (1.1)	45.8	CBCL	Anxiety/Depressive Symptoms, Internalizing Problems, Withdrawal
Klein et al. (2017)	Feasibility Study	40	6.8 (1.6)	62.0	CBCL, EVT-2, PPVT-4, TNL	Deficits in Oral Narration, Withdrawal
Bunnell et al. (2018)	Feasibility Study	15	9.6 (3.9)	/	Behavioral Assessment, Electrodermal Activity (EDA), Heart-Rate Variability (HRV, SPAI-C, SPAIC-PV	Anxiety, Social Anxiety
Starke (2018)	Cohort Study	18	/	/	BAV 3-11, TROG-D	Anxiety
Xu et al. (2018)	Pilot Study	12	/	75.0	Controlled Play Paradigm	Reduced Conversational Turns, Reduced Vocalization, Shorter Vocalization Duration
Catchpole et al. (2019)	Feasibility Study	31	6.5 (1.7)	51.6	EVT-2, PPVT-4, SCARED-PV, SNAP	Anxiety
Cornacchio et al. (2019)	Randomized Controlled Trial	29	6.6 (1.4)	75.9	ADIS-P, CBCL	Anxiety, Social Anxiety
Klein et al. (2019)	Cross-Sectional	42	7.1 (2.4)	47.6	BASC-3; EVT-2, PPVT-4, Test of Auditory Processing Skills, TNL	Impaired Functional Communication, Impaired Social Skills, Withdrawal
Longobardi et al. (2019)	Case-Control	/	7.6 (1.9)	/	Peer Nomination Questionnaire, SDQ, STRS, Y-CATS	Emotional Symptoms, Reduced Teacher-Perceived Closeness
Marotta et al. (2019)	Case-Control	30	9.5 (1.3)	56.7	SDSC	Sleep Problems
Milic et al. (2020)	Case-Control	25	5.7 (1.2)	80.0	ADIS-IV-C/P, Behavioral Observation Tasks, CBS, CSPS, DP-3, PCI, PPVT-IV, SASC-R/P, SDQ, STSC, TRSSA	Behavioral Inhibition, Emotional Symptoms, Fear of Negative Evaluation, Fewer Friendships/Difficulties in Friendship Formation, Shyness, Social Anxiety, Social Avoidance, Withdrawal

Melfsen et al. (2021)	Case-Control	28	12.7 (4.0)	64.3	A-DES, CDC, HSPS, SPAI-C	Sensory-Processing Sensitivity, Social Anxiety
Olivares-Olivares et al. (2021)	Cross-Sectional	110	6.6 (2.0)	64.5	CDI-S-P, MASC, MASC-P-ASO, MASC-P-ED, MASC-P-SF, SASC-R, SASC-R-FNE, SASC-R-SADG, SASC-R-SADN	Anxiety, Depressive Symptoms, Fear of Negative Evaluation, Harm Avoidance, Somatic Symptoms, Social Anxiety, Avoidance
Poole et al. (2021)	Case-Control	48	7.9 (0.5)	52.1	Salivary Cortisol, SCARED, SSRS, Videotaped Self-Presentation Task (Behavioral Observation)	State Social Anxiety, Trait Social Anxiety
Schwenck et al. (2021)	Quasi-Experiment	52	13.1 (3.5)	75.0	SPAI-C	Social Anxiety
Vogel & Schwenck (2021)	Case-Control	31	9.1 (1.2)	61.3	Psychophysiological Measurement: Heart Rate (HR), Skin Conductance Level (SCL), Respiratory Sinus Arrhythmia (RSA), SPAI-C	Inflexible Physiological Stress Response, Social Anxiety
Haggerty et al. (2022)	Feasibility Study	25	/	80.0	Daily Behavior Report, Observations of Speaking Behavior, SCARED	Anxiety
Melfsen et al. (2022)	Case-Control	28	12.7 (4.0)	64.3	FEEL-KJ, SPAI-C	Maladaptive Emotion Regulation
Vogel, Gensthaler, et al. (2022)	Cross-Sectional	28	9.4 (1.2)	64.3	Eye Tracking Paradigm, FSSM, SPAI-C	Freezing, Reduced Visual Exploration, Social Anxiety
Manti et al. (2022)	Cross-Sectional	28	4.5 (0.7)	61.0	CBCL, CSHQ	Anxiety, Internalizing Problems, Pervasive Developmental Problems, Sleep Problems
Shorer, Ben-Haim, Krispin, et al. (2023)	Cross-Sectional	78	5.8 (1.3)	67.9	Behavioral Observation, SASC-R	Social Anxiety
(Shorer, Ben-Haim, Klauzner, et al., 2023)	Feasibility Study	40	6.9 (2.1)	76.0	SASC-R	Social Anxiety
(de Jonge et al., 2024)	Cross-Sectional	34	13.2 (2.7)	73.5	Perception and Use of Computer-Mediated Communication	Reduced Use of Computer Mediated Communication
(Slobodin et al., 2024)	Cross-Sectional	78	/	/	SASC-R	Social Anxiety
(Slobodin et al., 2025)	Cross-Sectional	66	5.1 (0.8)	57.2	CBCL, SASC-R	Social Anxiety

* The data refer to the SM sample. ** Only those assessment instruments are listed for which symptom-related outcomes were reported in the respective study.

A-DES = Adolescent Dissociative Experience Scale; ADIS = Anxiety Disorders Interview Schedule; ASEBA = Achenbach System of Empirically Based Assessment; BASC-3 = Behavior Assessment System for Children; BAT = Behavioral Assessment Task; BAV 3-11 = Bochum Anxiety Measure for Preschool- and School-Age Children; BSI = Brief Symptom Inventory; BVRT = Benton Visual Retention Test; CBCL = Child Behavior Checklist; CBS = Child Behavior Scale; CCC-2 = Children's Communication Checklist; CDC = Child Dissociative Checklist; CDI = Children's Depression Inventory; CFT 20-R = Culture Fair Intelligence Test; CPRS-R:L = Conners' Parent Rating Scale–Revised; CRS-R = Conners' rating scale-revised; CRS–R = Conners Rating Scales—Revised; CSHQ = Children's Sleep Habits Questionnaire; CSI-4 = Child Symptom Inventory-4; CSPS = Child Social Preference Scale; CTRS-R:L = Conners' Teacher Rating Scale–Revised; DISYPS-III = Diagnostic System for Mental Disorders in Childhood and Adolescence; DP-3 = Developmental Profile 3; EAS = Emotionality, Activity, and Sociability (EAS) Temperament Survey; ECBI = Eyberg Child Behavior Inventory; EVT-2 = Expressive Vocabulary Test–2; FEEL-KJ = Emotion Regulation Strategies Questionnaire; FSSC-R = Fear Survey Schedule for Children-Revised; FSSM = Frankfurt Scale of Selective Mutism; HSPS = Highly Sensitive Person Scale; LACT = Lindamood Auditory Conceptualization Test; MASC = Multidimensional Anxiety Scale for Children; OCHS-R = Revised Ontario Child Health Study Scales; PCI = Play Choice Interview; PPVT-III = Peabody Picture Vocabulary Test III; RCMAS = Revised

Children's Manifest Anxiety Scale; SASC-R = Social Anxiety Scale for Children – Revised; SCARED = Screen for Child Anxiety Related Emotional Disorders; SCAS = Spence Children's Anxiety Scale; SCQ = Social Communication Questionnaire; SDSC = Sleep Disturbance Scale for Children; SDQ = Strengths and Difficulties Questionnaire; SDQ-P = Strengths and Difficulties Questionnaire – Parent; SDQ-T = Strengths and Difficulties Questionnaire – Teacher; SNAP = Strong Narrative Assessment Procedure; SPAI-C = Social Phobia and Anxiety Inventory for Children; SPAIC-PV = Social Phobia and Anxiety Inventory for Children-Parent Version; SRS = Social Responsiveness Scale; SSQ = School Speech Questionnaire; SSRS = Social Skills Rating System; STAI-C = State-Trait Anxiety Inventory for Children Trait subscale; STRS = Student–Teacher relationship scale; STSC = Short Temperament Scale for Children; TNL = Test of Narrative Language; TRF = Teacher Report Form; TROG = Test of Reception of Grammar; TRSSA = Teacher Rating Scale for Social Anxiety; VABS–II = Vineland-II Adaptive Behavior Scales; WISC = Wechsler Intelligence Scales; WRAT-R = Revised Wide Range Achievement Test; Y-CATS = Young Children's Appraisal of Teacher Support; YSR = Youth Self Report.

Table S2. Characteristics of Studies without Normative Reference.

Study	Study Design	Sample Characteristics*			Symptom Measures	Symptoms Identified as Clinically Relevant
		N	Mean Age (SD)	% female		
Wergeland, H. (1979)	Cohort Study	11	/	64	Medical Case Reports	Aggression, Anxiety, Obstinacy, Pathological EEG, Reduced Contact Function/Contact Problems, Stubborn, Submissive
Hayden, T. L. (1980)	Case Series	66	/	84.8	Diverse Information Sources: Direct Observations, Video-/Audiotapes, Written Reports, Parent, Teacher and Pediatrician Questionnaires, Other Written Material (Psychological, Psychiatric, Pediatric Reports)	Aberrant Motor Activity, Aggression, Antisocial Behavior, Clinging, Fearfulness, Frequent Crying, Hostility, Immaturity, Lack of Facial Expression, Lack or Appropriate Affect, Manipulativeness, Negativism, Obsessive-Compulsive Behaviors, Passivity, Phobias, Physical Tension, Poor Eating, Poor Sleeping, Sensitivity, Shyness, Stubbornness, Submissiveness, Temper Tantrums, Timidity, Withdrawal
Wilkins, R. (1985)	Case-Control	24	/	71.0	Medical Case Reports	Anxiety, Depressive Symptoms, Jealousy, Manipulativeness, Over-Dependence, Shyness, Temper Tantrums
Krohn, D. et al. (1992)	Case Series	20	/	60.0	Medical Case Reports	Controlling, Negativism, Oppositionality/Defiance
Steinhausen, H. C. & Juzi, C. (1996)	Case Series	100	8.7 (3.4)	61.0	CBCL (Subsample), Clinical Rating, Item Sheets Designed by the Authors, Medical Case Reports	Anxiety, Anxiety/Depressive Symptoms, Attention Problems, Hyperactivity, Internalizing Problems, Obsessions/Compulsions, Oppositionality/Defiance/Aggression, Shyness, Social Problems, Thought Problems, Withdrawal
Brix Andersson, C., & Hove Thomsen, P. (1998)	Case-Control	37	/	46.0	Medical Case Reports	Aggression, Articulation Difficulties, Pathological EEG, Sensitivity, Shyness, Stubbornness, Sulkiness, Sulkiness, Weepiness
Kumpulainen, K. et al. (1998)	Cross-Sectional	38	/	61.0	Teacher Report Questionnaire Developed by the Authors	Aggression, Dependency, Expressionlessness, Hyperkinetic, Immaturity, Language Deficits, Seriousness, Shyness, Withdrawal
Remschmidt, H. et al. (2001)	Cohort Study	45	8.7 (3.6)	48.9	Clinician-Rated Assessment, Standardized Documentation Sheet	Anxiety, Articulation Difficulties, Depressive Symptoms, Dysphoric Mood, Fear of Speaking,

						Impulsivity, Insecurity/Self-Esteem Impairments, Irritability, Motor Problems, Poor Concentration, Reduced Contact Function/Contact Problems, Somatic Symptoms
Schwartz, R. H. et al. (2006)	Cross-Sectional	33	7.9	72.7	Survey designed by the Authors	Communication Problems, Sensory Integration Problems, Shyness
Omdal, H., & Galloway, D. (2008)	Qualitative	11	/	/	Semi-structured Parent Interviews; Video-Observations of the Children's Social Interactions	Avoidance, Determination/Stubbornness, Fear of Speaking, Feelings of Loneliness, Self-Consciousness, Social Anxiety, Social Withdrawal
Edison, S. C. et al. (2011)	Cross-Sectional	21	/	62.0	Observational Rating in Free Play and Social Task, SCARED-P	Anxiety, Reduced Speaking Behavior, Reduced Spontaneous Speaking
Steffenburg, H. et al. (2018)	Case Series	97	/	73.2	Medical Case Reports	Avoidance, Shyness
Vogel, F. et al. (2019)	Qualitative	51	13.1 (3.5)	76.5	FSSM, Online Survey: Open-Ended Question Regarding Fears, SPAI-C	Anxiety, Fear, Social Anxiety
Williams, C. E. et al. (2021)	Qualitative	/	/	/	Semi-structured Teacher Interview	Anxiety, Manipulativeness/Stubbornness
Schwenck, C. et al. (2022)	Cross-Sectional	91	8.0 (3.9)	74.7	Open-ended Questions about Anxiety-Inducing Stimuli	Anxiety
Dogru, H. et al. (2023)	Cross-Sectional	49	7.6 (2.3)	63.0	Medical Case Reports	Poor Peer Relationships, School Refusal, Shyness, Withdrawal or Fear of Social Embarrassment
Freitag, G. F. et al. (2024)	Cross-Sectional	152	6.1 (2.1)	67.1	ARI-P	Phasic Irritability, Tonic Irritability
Keville, S. et al. (2024)	Qualitative	12	12.7 (4.6)	41.7	Semi-structured Parent Interview	Anxiety, Avoidance, Communication Freezes, Fatique, Fear, Introversion, Loneliness, Sensory Sensitivity, Shutdown, Suicidality, Worries
Vogel, F. et al. (2024)	Cross-Sectional	86	9.7 (4.4)	66.0	DISYPS-III FBB-SOZ, FSSM, Open Response Parent Questionnaire	Avoidance/Safety Behavior, Externalizing Behavior, Fear, Freezing, Negative Affect, Reassuring Behavior, Reduced Body Tension and Slackness, Reduced Communication, Regressive Behavior or Displacement Activity, Self-Esteem Impairment, Whining

* The data refer to the SM sample. ** Only measures for which results were reported in the corresponding study are mentioned.

ARI-P = Affective Reactivity Index; CBCL = Child Behavior Checklist; DISYPS-III = Diagnostic System for Mental Disorders in Childhood and Adolescence; FSSM = Frankfurt Scale of Selective Mutism; SCARED = Screen for Child Anxiety Related Emotional Disorders; SPAI-C = Social Phobia and Anxiety Inventory for Children.

Supplementary S2

Table S3. Symptom Clusters and Included Features (Studies with Normative Reference).

Symptom Cluster	Symptoms Encompassed
Anxiety	• Anxiety<br>• Generalized Anxiety<br>• Nervousness
Attention Deficits	• Attention Deficit/Hyperactivity<br>• Attention Problems<br>• Inattention<br>• Hyperactivity/Impulsivity
Auditory Processing Deficits	• Differential Auditory Processing<br>• Specific Deficiencies in Auditory Efferent Activity
Behavioral Inhibition	• Behavioral Inhibition<br>• Reduced Visual Exploration
Deficits in Social Cognition and Awareness	• Reduced Social Responsibility<br>• Social Awareness Problems<br>• Social Cognition Problems<br>• Social Motivation Problems
Depressive Symptoms	• Depressive Symptoms
Emotion Regulation Difficulties	• Affective Problems<br>• Emotional Difficulties<br>• Emotional Lability<br>• Emotional Symptoms<br>• Emotionality<br>• Maladaptive Emotion Regulation
Externalizing and Behavioral Problems	• Aggression<br>• Antisocial Behavior<br>• Behavioral Problems<br>• Conduct Problems<br>• Externalizing Behavior<br>• Oppositional Behavior
Freezing and Performance Problems	• Freezing<br>• Problems with Public Performance
Harm Avoidance	• Harm Avoidance
Impaired Social Interaction Skills	• Impaired Social Skills/Social Problems<br>• Reduced Nonverbal Cooperation<br>• Reduced Nonverbal Social Skills<br>• Reduced Social Assertion<br>• Reduced Social Cooperation<br>• Reduced Social Skills<br>• Reduced Verbal Social Skills<br>• Social Communication Problems
Impaired Social/Peer Relationships	• Fewer Friendships/Difficulties in Friendship Formation<br>• Lower Peer Acceptance<br>• Peer Problems<br>• Problems with Peer Interaction<br>• Reduced Teacher-Perceived Closeness<br>• Social Problems
Inflexible Physiological Stress Response	• Cortisol Stress Reactivity<br>• Increased Tonic Arousal<br>• Reduced Reactivity/Inflexible Stress Response
Internalizing Problems	• Internalizing Problems
Irritability and Stubbornness	• Irritability<br>• Stubbornness
Language/Communication Impairments (Expressive)	• Communication Problems: Speech<br>• Communication Problems: Syntax<br>• Deficits in Oral Narration<br>• Reduced Expressive Narrative Language Competence<br>• Reduced Expressive Vocabulary<br>• Syntax Problems
Language/Communication Impairments (General)	• Impaired Functional Communication<br>• Language Impairments

Lower Self-Control	• Lower Self Control
Memory Deficits	• Reduced Auditory-Verbal Memory Span<br>• Visual Memory Deficits
Motor Skill Impairments	• Motor Skill Impairments
Pervasive Developmental Problems	• Pervasive Developmental Problems
Reduced Joint Attention	• Reduced Joint Attention
Reduced Social Engagement and Participation	• Reduced Activity Levels<br>• Reduced Social Activity<br>• Reduced Social Engagement
Reduced Verbal Participation	• Reduced Conversational Turns<br>• Reduced Use of Computer Mediated Communication<br>• Reduced Vocalization<br>• Shorter Vocalization Duration
Repetitive and Compulsive Behaviors	• Autistic Mannerisms<br>• Obsessional<br>• Obsessive-Compulsive Behaviors<br>• Perfectionism<br>• Tics/Stereotypies
Separation Anxiety	• Separation Anxiety
Sensory-Processing Sensitivity	• Sensory-Processing Sensitivity
Shyness	• Shyness
Sleep Problems	• Sleep Problems
Social and Situational Avoidance	• Avoidance of Verbal Situations<br>• School Avoidance<br>• Social Avoidance
Social Anxiety	• Fear of Negative Evaluation<br>• Social Anxiety<br>• State Social Anxiety<br>• Trait Social Anxiety
Somatic Complaints	• Somatic Complaints<br>• Somatic Symptoms
Specific Fears	• Agoraphobic Fears<br>• Fear of Animals<br>• Fear of Danger<br>• Fear of Medical<br>• Fear of Speaking<br>• Specific Fears
Thought Problems	• Thought Problems
Withdrawal	• Temperament Withdrawal<br>• Withdrawal

Table S4. Symptom Clusters and Included Features (Studies without Normative Reference).

**Symptom Cluster**	**Symptoms Encompassed**
Anxiety	• Anxiety<br>• Attentional Focus on Bodily Anxiety Symptoms
Attention Deficits	• Attention Problems<br>• Poor Concentration
Attentional Focus on Thoughts	• Attentional Focus on Thoughts
Body Tension Problems	• Reduced Body Tension<br>• Rigid, Tense Posture
Communication Problems	• Articulation Difficulties<br>• Communication Freezing<br>• Language/Speech Deficit<br>• Reduced Communication<br>• Reduced Spontaneous Speaking<br>• Reduced Verbal Communication
Controlling Behavior	• Controlling Behavior
Crying Behaviors	• Frequent Crying<br>• Weepiness<br>• Whining
Depressive Symptoms	• Depressive Symptoms<br>• Feelings of Loneliness<br>• Suicidality
Eating Disturbances	• Eating Disturbances

Emotional Expressionlessness	• Expressionlessness<br>• Lack of Affect
Externalizing and Behavioral Problems	• Aggression<br>• Antisocial Behavior<br>• Externalizing Behavior<br>• Hostility/Anger<br>• Oppositional Behavior<br>• Stubbornness<br>• Temper Tantrums
Fatigue	• Fatigue
Fear of Mistakes	• Fear of Mistakes
Freezing and Shutdown	• Freezing<br>• Shutdown
Hyperactivity/Impulsivity	• Impulsivity<br>• Hyperactivity
Immaturity and Regressive Behavior	• Immaturity<br>• Regressive Behavior
Insecurity/Dependency	• Avoidance of Eye Contact<br>• Clinginess<br>• Over-Dependency<br>• Reassurance-Seeking Behavior
Internalizing Problems	• Internalizing Problems
Introversion	• Introversion
Irritability	• Irritability<br>• Phasic Irritability<br>• Tonic Irritability
Jealousy	• Jealousy
Language-/Voice-Related Fears	• Language-Related Fears<br>• Voice-Related Fears
Manipulativeness	• Manipulativeness
Mood Problems	• Dysphoric Mood<br>• Sulkiness
Motor Problems	• Motor Problems<br>• Unusual Motor Activity
Negativity	• Negativity
Pathological EEG	• Pathological EEG
Reduced Contact Function/Contact Problems	• Reduced Contact Function/Contact Problems
Repetitive and Compulsive Behaviors	• Obsessions/Compulsions<br>• Ritualistic, Compulsive Behaviors
Self-Consciousness and Self-Esteem Issues	• Impaired Self-Esteem<br>• Self-Consciousness
Sensitivity	• Sensitivity
Sensory Problems	• Sensory Problems/Sensory Processing Disorder<br>• Sensory-Processing Sensitivity
Seriousness	• Seriousness
Shyness	• Shyness
Sleep Problems	• Sleep Problems
Social and Situational Avoidance	• School Avoidance<br>• Social Avoidance
Social Anxiety	• Fear of Social Embarrassment<br>• Social Anxiety
Social Problems	• Problems with Peer Interaction<br>• Social Problems
Somatic Complaints	• Somatic Complaints
Specific Fears/General Fear	• Fear<br>• Fear of Speaking
Submissiveness	• Submissiveness
Thought Problems	• Thought Problems
Withdrawal	• Social Withdrawal<br>• Withdrawal
Worries	• Worries

Supplementary S3

Table S5. Quality Assessment of Case-Control Studies.

Study	Quality Domains									
	D1	D2	D3	D4	D5	D6	D7	D8	D9	D10
Wilkins (1985)	Y	Y	?	N	Y	?	?	N	Y	Y
Brix Andersson & Hove Thomsen (1998)	?	Y	Y	?	?	?	0	?	Y	Y
Kristensen (2001)	Y	Y	?	Y	Y	Y	Y	Y	/	Y
Kristensen (2002)	Y	Y	Y	?	Y	Y	?	?	/	Y
Kristensen & Torgersen (2002)	Y	Y	?	Y	Y	N	N	Y	/	Y
Elizur & Perednik (2003)	Y	Y	Y	Y	Y	?	?	Y	/	Y
Manassis et al. (2003)	Y	?	Y	Y	Y	N	N	Y	Y	Y
Yeganeh et al. (2003)	Y	Y	Y	Y	Y	N	N	Y	/	Y
Bar-Haim et al. (2004)	Y	Y	?	Y	Y	N	N	Y	/	Y
Cunningham et al. (2004)	Y	Y	?	Y	Y	?	?	Y	/	Y
McInnes et al. (2004)	Y	Y	?	Y	Y	?	?	Y	/	Y
Vecchio & Kearney (2005)	Y	Y	Y	Y	Y	?	?	Y	/	Y
Cunningham et al. (2006)	Y	Y	Y	Y	Y	?	?	Y	/	Y
Kristensen & Oerbeck (2006)	Y	Y	Y	Y	Y	?	?	Y	/	Y
Melfsen et al. (2006)	?	?	Y	Y	Y	N	N	Y	/	Y
Yeganeh et al. (2006)	Y	?	Y	Y	Y	?	?	Y	/	Y
Arie et al. (2007)	Y	?	Y	Y	Y	N	N	Y	/	Y
Manassis et al. (2007)	Y	?	Y	Y	Y	Y	Y	Y	/	Y
Bergman et al. (2008)	Y	?	Y	Y	Y	N	N	Y	/	Y
Oerbeck & Kristensen (2008)	Y	Y	Y	Y	?	Y	Y	Y	/	Y
Carbone et al. (2019)	Y	Y	Y	Y	Y	Y	Y	Y	/	Y
Henkin et al. (2010)	Y	Y	Y	Y	Y	N	N	Y	Y	Y
Nowakowski et al. (2011)	Y	?	Y	Y	Y	?	?	Y	/	Y
Heilman et al. (2012)	Y	?	Y	Y	Y	?	?	Y	Y	Y
Young et al. (2012)	N	N	Y	/	/	N	?	Y	/	?
Alyanak et al. (2013)	Y	?	/	Y	Y	?	?	Y	/	Y
Levin-Decanini et al. (2013)	Y	?	Y	Y	Y	?	?	Y	/	Y
Muchnik et al. (2013)	Y	Y	Y	Y	Y	Y	Y	Y	/	Y
Cholemkery et al. (2014)	Y	Y	Y	Y	Y	Y	Y	Y	/	Y
Martinez et al. (2015)	Y	?	Y	Y	Y	Y	N	Y	/	Y
Gensthaler et al. (2016)	Y	Y	Y	Y	Y	?	?	Y	/	Y
Longobardi et al. (2019)	Y	Y	Y	Y	Y	N	N	Y	/	Y
Marotta et al. (2019)	Y	Y	Y	Y	Y	N	N	Y	/	Y
Milic et al. (2020)	Y	?	Y	Y	Y	Y	Y	Y	/	Y
Melfsen et al. (2021)	Y	?	Y	Y	Y	Y	Y	Y	/	Y
Poole et al. (2021)	Y	?	Y	Y	Y	Y	Y	Y	/	Y
Vogel & Schwenck (2021)	Y	Y	Y	Y	Y	Y	Y	Y	/	Y
Melfsen et al. (2022)	Y	Y	Y	Y	Y	Y	Y	Y	/	Y

Note. D1 = Were the groups comparable other than the presence of disease in cases or the absence of disease in controls?; D2 = Were cases and controls matched appropriately?; D3 = Were the same criteria used for identification of cases and controls?; D4 = Was exposure measured in a standard, valid and reliable way?; D5 = Was exposure measured in the same way for cases and controls?; D6 = Were confounding factors identified?; D7 = Were strategies to deal with confounding factors stated?; D8 = Were outcomes assessed in a standard, valid and

reliable way for cases and controls?; D9 = Was the exposure period of interest long enough to be meaningful?; D10 = Was appropriate statistical analysis used?; Y = Yes; N = No; ? = Unclear/Partially; / = Not Applicable.

Table S6. Quality Assessment of Cross-Sectional Studies.

Study	Quality Domains							
	D1	D2	D3	D4	D5	D6	D7	D8
Black & Uhde (1995)	Y	Y	Y	Y	?	N	Y	Y
Dummit et al. (1997)	Y	Y	Y	Y	?	N	Y	Y
Kumpulainen et al. (1998)	Y	Y	?	?	?	N	N	Y
Schwartz et al. (2006)	Y	Y	N	Y	N	N	N	Y
Cohan et al. (2008)	Y	Y	Y	Y	?	N	Y	Y
Letamendi et al. (2008)	Y	Y	Y	Y	Y	Y	Y	Y
Edison et al. (2011)	Y	?	Y	Y	Y	Y	Y	Y
Klein et al. (2013)	Y	Y	Y	Y	N	N	Y	Y
Klein et al. (2019)	Y	Y	Y	Y	?	N	Y	Y
Olivares-Olivares et al. (2021)	Y	Y	Y	Y	?	N	Y	Y
Schwenck et al. (2022)	Y	Y	?	Y	Y	N	Y	Y
Vogel et al. (2022)	Y	Y	Y	Y	/	/	Y	Y
Manti et al. (2022)	Y	Y	Y	Y	Y	Y	Y	Y
Dogru et al. (2023)	Y	Y	?	Y	N	N	Y	Y
Shorer et al. (2023)	Y	Y	Y	Y	?	?	Y	Y
De Jonge et al. (2024)	Y	Y	Y	Y	?	?	Y	Y
Freitag et al. (2024)	Y	Y	Y	Y	Y	Y	Y	Y
Slobodin et al. (2024)	Y	Y	Y	Y	Y	Y	Y	Y
Vogel et al. (2024)	Y	Y	Y	Y	?	?	Y	Y
Slobodin et al. (2025)	Y	Y	Y	Y	?	N	Y	Y

Note. D1 = Were the criteria for inclusion in the sample clearly defined?; D2 = Were the study subjects and the setting described in detail?; D3 = Was the exposure measured in a valid and reliable way?; D4 = Were objective, standard criteria used for measurement of the condition?; D5 = Were confounding factors identified?; D6 = Were strategies to deal with confounding factors stated?; D7 = Were the outcomes measured in a valid and reliable way?; D8 = Was appropriate statistical analysis used?; Y = Yes; N = No; ? = Unclear/Partially; / = Not Applicable.

Table S7. Quality Assessment of Quasi-Experimental Studies.

Study	Quality Domains								
	D1	D2	D3	D4	D5	D6	D7	D8	D9
Dummit et al. (1996)	Y	N	/	/	Y	Y	Y	Y	Y
Oerbeck et al. (2012)	Y	N	/	Y	Y	Y	Y	Y	Y
Klein et al. (2017)	Y	N	/	/	Y	Y	Y	Y	Y
Bunnell et al. (2018)	Y	N	/	/	Y	Y	Y	Y	Y
Xu et al. (2018)	Y	N	/	/	Y	Y	Y	Y	Y
Catchpole et al. (2019)	Y	N	/	/	Y	Y	Y	Y	Y
Schwenck et al. (2021)	/	Y	Y	/	Y	Y	Y	/	Y
Haggerty et al. (2022)	Y	N	/	/	Y	Y	Y	N	Y
Shorer et al. (2023)	Y	N	/	/	Y	Y	Y	?	Y

Note. D1 = Is it clear in the study what is the "cause" and what is the "effect"?; D2 = Was there a control group?; D3 = Were participants included in any comparisons similar?; D4 = Were the participants included in any comparisons receiving similar treatment/care, other than the exposure or intervention of interest?; D5 = Were there multiple measurements of the outcome, both pre and post the intervention/exposure?; D6 = Were the outcomes of participants included in any comparisons measured in the same way?; D7 = Were outcomes measured in a reliable way?; D8 = Was follow-up complete and if not, were differences between groups in terms of their

follow-up adequately described and analyzed?; D9 = Was appropriate statistical analysis used?; Y = Yes; N = No; ? = Unclear/Partially; / = Not Applicable.

Table S8. Quality Assessment of Randomized Controlled Trials.

Study	Quality Domains												
	D1	D2	D3	D4	D5	D6	D7	D8	D9	D10	D11	D12	D13
Esposito et al. (2017)	Y	?	Y	N	N	Y	N	Y	Y	Y	N	Y	Y
Cornacchio et al. (2019)	Y	?	Y	N	N	Y	Y	Y	Y	Y	Y	Y	Y

Note. D1 = Was true randomization used for assignment of participants to treatment groups?; D2 = Was allocation to treatment groups concealed?; D3 = Were treatment groups similar at the baseline?; D4 = Were participants blind to treatment assignment?; D5 = Were those delivering the treatment blind to treatment assignment?; D6 = Were treatment groups treated identically other than the intervention of interest?; D7 = Were outcome assessors blind to treatment assignment?; D8 = Were outcomes measured in the same way for treatment groups?; D9 = Were outcomes measured in a reliable way?; D10 = Was follow up complete and if not, were differences between groups in terms of their follow up adequately described and analyzed?; D11 = Were participants analyzed in the groups to which they were randomized?; D12 = Was appropriate statistical analysis used?; D13 = Was the trial design appropriate and any deviations from the standard RCT design accounted for in the conduct and analysis of the trial?; Y = Yes; N = No; ? = Unclear/Partially; / = Not Applicable.

Table S9. Quality Assessment of Case Series.

Study	Quality Domains									
	D1	D2	D3	D4	D5	D6	D7	D8	D9	D10
Hayden (1980)	Y	Y	Y	?	?	Y	Y	?	Y	?
Krohn et al. (1992)	Y	?	Y	?	?	Y	Y	Y	Y	Y
Steinhausen & Juzi (1996)	Y	Y	Y	?	?	Y	Y	?	Y	Y
Steffenburg et al. (2018)	Y	Y	Y	?	Y	Y	Y	?	Y	Y

Note. D1 = Were there clear criteria for inclusion in the case series?; D2 = Was the condition measured in a standard, reliable way for all participants?; D3 = Were valid methods used for identification of the condition for all participants?; D4 = Did the case series have consecutive inclusion of participants?; D5 = Did the case series have complete inclusion of participants?; D6 = Was there clear reporting of the demographics of the participants in the study?; D7 = Was there clear reporting of clinical information of the participants?; D8 = Were the outcomes or follow up results clearly reported?; D9 = Was there clear reporting of the presenting site(s)/clinic(s) demographic information?; D10 = Was appropriate statistical analysis used?; Y = Yes; N = No; ? = Unclear/Partially; / = Not Applicable.

Table S10. Quality Assessment of Cohort Studies.

Study	Quality Domains										
	D1	D2	D3	D4	D5	D6	D7	D8	D9	D10	D11
Wergeland (1997)	/	/	Y	Y	N	/	?	Y	?	Y	?
Remschmidt et al. (2001)	?	/	Y	Y	?	/	Y	Y	Y	Y	Y
Sharkey & McNicholas (2012)	/	/	Y	?	N	/	Y	/	/	/	Y
Starke (2018)	Y	Y	Y	Y	?	/	Y	Y	?	N	Y

Note. D1 = Were the two groups similar and recruited from the same population?; D2 = Were the exposures measured similarly to assign people to both exposed and unexposed groups?; D3 = Was the exposure measured in a valid and reliable way?; D4 = Were confounding factors identified?; D5 = Were strategies to deal with confounding factors stated?; D6 = Were the groups/participants free of the outcome at the start of the study (or at the moment of exposure)?; D7 = Were the outcomes measured in a valid and reliable way?; D8 = Was the follow up time reported and sufficient to be long enough for outcomes to occur?; D9 = Was follow up complete, and if not, were the reasons to loss to follow up described and explored?; D10 = Were strategies to address

incomplete follow up utilized?; D11 = Was appropriate statistical analysis used?; Y = Yes; N = No; ? = Unclear/Partially; / = Not Applicable.

Table S11. Quality Assessment of Qualitative Studies.

Study	Quality Domains									
	**D1**	**D2**	**D3**	**D4**	**D5**	**D6**	**D7**	**D8**	**D9**	**D10**
Omdal & Galloway (2008)	/	Y	Y	Y	Y	N	N	Y	Y	Y
Vogel et al. (2019)	/	Y	Y	Y	Y	N	N	Y	Y	Y
Williams et al. (2021)	/	Y	Y	Y	Y	?	?	Y	Y	Y
Keville et al. (2024)	/	Y	Y	Y	Y	?	Y	Y	Y	Y

Note. D1 = Is there congruity between the stated philosophical perspective and the research methodology?; D2 = Is there congruity between the research methodology and the research question or objectives?; D3 = Is there congruity between the research methodology and the methods used to collect data?; D4 = Is there congruity between the research methodology and the representation and analysis of data?; D5 = Is there congruity between the research methodology and the interpretation of results?; D6 = Is there a statement locating the researcher culturally or theoretically?; D7 = Is the influence of the researcher on the research, and vice versa, addressed?; D8 = Are participants, and their voices, adequately represented?; D9 = Is the research ethical according to current criteria or is there evidence of ethical approval by an appropriate body?; D10 = Do the conclusions drawn in the research report flow from the analysis or interpretation of the data?; Y = Yes; N = No; ? = Unclear/Partially; / = Not Applicable.

Table S12. Quality Assessment of Prevalence Studies.

Study	Quality Domains								
	**D1**	**D2**	**D3**	**D4**	**D5**	**D6**	**D7**	**D8**	**D9**
Bergman et al. (2002)	Y	Y	Y	Y	Y	Y	Y	Y	Y

Note. D1 = Was the sample frame appropriate to address the target population?; D2 = Were study participants sampled in an appropriate way?; D3 = Was the sample size adequate?; D4 = Were the study subjects and the setting described in detail?; D5 = Was the data analysis conducted with sufficient coverage of the identified sample?; D6 = Were valid methods used for the identification of the condition?; D7 = Was the condition measured in a standard, reliable way for all participants?; D8 = Was there appropriate statistical analysis?; D9 = Was the response rate adequate, and if not, was the low response rate managed appropriately?; Y = Yes; N = No; ? = Unclear/Partially; / = Not Applicable.
